# Multimodal floral cues guide mosquitoes to tansy inflorescences

**DOI:** 10.1038/s41598-019-39748-4

**Published:** 2019-03-07

**Authors:** Daniel A. H. Peach, Regine Gries, Huimin Zhai, Nathan Young, Gerhard Gries

**Affiliations:** 1 Department of Biological Sciences, Simon Fraser University, Burnaby, British Columbia V5A 1S6 Canada; 2 Present Address: Eurofins|Alphora Research Inc., Mississauga, Ontario, L5K 1B3 Canada

**Keywords:** Chemical ecology, Behavioural ecology

## Abstract

Female mosquitoes exploit olfactory, CO_2_, visual, and thermal cues to locate vertebrate hosts. Male and female mosquitoes also consume floral nectar that provides essential energy for flight and survival. Heretofore, nectar-foraging mosquitoes were thought to be guided solely by floral odorants. Using common tansies, *Tanacetum vulgare* L., northern house mosquitoes, *Culex pipiens* L., and yellow fever mosquitoes, *Aedes aegypti* (L.), we tested the hypothesis that the entire inflorescence Gestalt of olfactory, CO_2_ and visual cues is more attractive to mosquitoes than floral odorants alone. In laboratory experiments, we demonstrated that visual and olfactory inflorescence cues in combination attract more mosquitoes than olfactory cues alone. We established that tansies become net producers of CO_2_ after sunset, and that CO_2_ enhances the attractiveness of a floral blend comprising 20 synthetic odorants of tansy inflorescences. This blend included nine odorants found in human headspace. The “human-odorant-blend” attracted mosquitoes but was less effective than the entire 20-odorant floral blend. Our data support the hypothesis that the entire inflorescence Gestalt of olfactory, CO_2_ and visual cues is more attractive to mosquitoes than floral odorants alone. Overlapping cues between plants and vertebrates support the previously postulated concept that haematophagy of mosquitoes may have arisen from phytophagy.

## Introduction

Females of many mosquito species require the nutrients obtained from a vertebrate blood meal for egg development. However, both male and female mosquitoes also consume plant sugars, primarily as floral nectar^1,2^, that provide essential energy for flight and survival^1–3^, thus enabling populations even of highly synanthropic mosquitoes to persist^3^. As pollinators^4,5^ or nectar thieves^6^, mosquitoes seek the inflorescences of many plant species^7,8^, responding to floral semiochemicals (message-bearing chemicals) that apparently guide them to floral resources^9^.

Mosquitoes use olfactory, visual, and thermal cues to locate vertebrate hosts, including humans. Important olfactory cues are CO_2_^10^, L-lactic acid^11^ and other carboxylic acids^12,13^. CO_2_ also attracts, or prompts host-seeking behaviour of, other haematophagous insects including tsetse flies (*Glossina* spp.), kissing bugs, biting midges (*Culicoides* spp.) and black flies (Diptera: Simuliidae)^14^. In mosquitoes, CO_2_ interacts with other host cues^15,16^; however, CO_2_ originates not only from vertebrate hosts but also from plants that emit CO_2_ as a metabolite of cellular respiration^17^. During diurnal photosynthesis, plants are net CO_2_ sinks but at dusk cease photosynthesis and become net CO_2_ producers, thus increasing ambient CO_2_ concentrations^17–19^. The plants’ transition from net CO_2_ sinks to net CO_2_ producers coincides with peak nectar foraging activity of many mosquito species^2,8^. Plant CO_2_ mediates insect attraction in many plant-insect interactions^14^ and serves as a foraging cue for nectar-feeding insects^20^ including the haematophagous sand fly, *Phlebotomus papatasi*^21^.

The role of visual inflorescence cues for mosquito attraction has barely been explored. Mosquitoes frequent mostly light-coloured inflorescences^1,2^, or dark inflorescence mimics in the presence of a human observer^22^, but the underlying mechanisms are not known^2,22^. Light-coloured and strongly-scented inflorescences are often pollinated by crepuscular or nocturnal moths^23–26^, and sometimes are co-pollinated by mosquitoes^27^. Interactive effects between visual and olfactory cues have been studied in plant-heteroceran systems^28,29^, as have been innate colour and odour preferences that experimentally can be manipulated via reward-based learning^30,31^. The olfactory cues of oxeye daisies, with or without intact visual cues, suffice to attract mosquitoes^32^ that learn to associate artificial visual cues with the nutrient quality of sugar rewards^33^. However, visual cues mediate other plant-pollinator interactions^34^, and guide host-foraging mosquitoes, provided they have been impelled by elevated levels of CO_2_^15^. The concept of CO_2_-“gated” activity may be applicable not only to host-foraging but also to nectar-foraging mosquitoes^35^, but remains to be studied in this context to fully understand the inflorescence cue complex.

Interestingly, some human-headspace semiochemicals (1-octen-3-ol, nonanal, specific carboxylic acids) attractive to host-seeking mosquitoes^13,36,37^ are also present in the odor bouquet of inflorescences frequented by nectar-foraging mosquitoes^9,38^. How frequently semiochemicals are shared by human host and plant resources remains unknown.

Various species of mosquitoes frequent the inflorescences of common tansies, *Tanacetum vulgare*^5,7,8^, likely in response to floral odor^1,2^. Working with the tansy-pollinating^5^ northern house mosquito, *Culex pipiens* L., and the yellow fever mosquito, *Aedes aegypti* (L.), as model species, we tested the hypotheses (1) that tansy-foraging females, analogous to human host-foraging females, exploit a multimodal complex of CO_2_, semiochemical and visual floral cues, and (2) that key floral semiochemicals are shared with human hosts.

## Results

### Effect of Olfactory and Visual Tansy Inflorescence Cues on Mosquito Attraction

**Figure 1:**
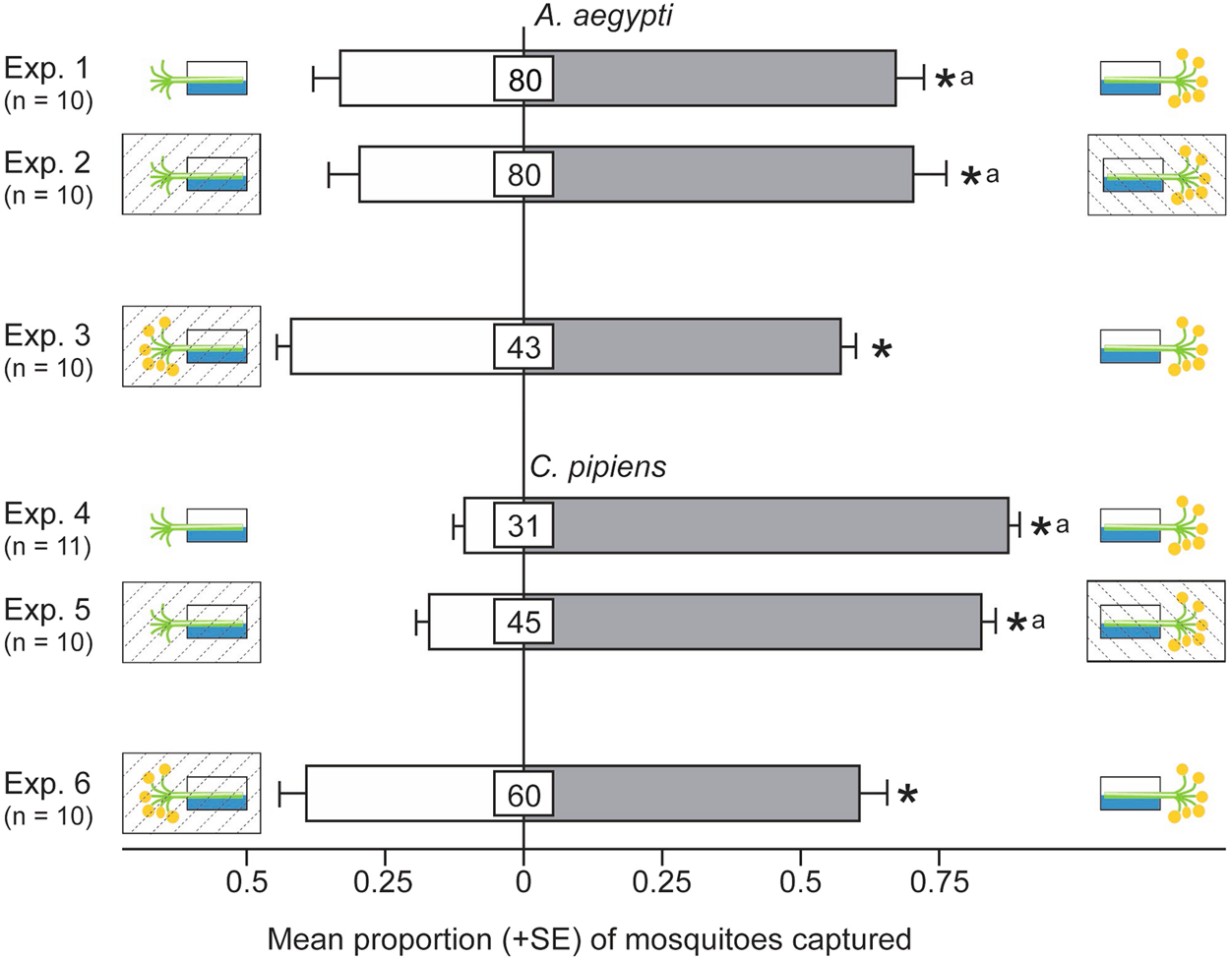
Effect of olfactory and visual tansy inflorescence cues on attraction of female *A*. *aegypti* and *C*. *pipiens*. Trap captures of 1- to 3-day-old female *A*. *aegypti* and 1- to 3-day-old female *C*. *pipiens* in response to visual and olfactory tansy inflorescence cues. A rectangular box with hatched lines indicates that the occluded inflorescence offered no visual cues. An asterisk indicates a significant preference (*P* < 0.01) for the specific test stimulus (binary logistic regression analyses with logit link function); the same letter on paired bars in parallel experiments indicates no difference in the mean proportion of mosquitoes responding to respective stimuli (*P* > 0.05); numbers within bars indicate the mean percentage of mosquitoes not captured.

In two-choice laboratory experiments with a paired-trap design, traps baited with a non-occluded (i.e., fully visible) inflorescence captured more female *A*. *aegypti* (z = 5.5, *P* < 0.0001) and *C*. *pipiens* (z = 12.8, *P* < 0.0001) than traps fitted with a non-occluded stem of an inflorescence (Fig. 1; Exps 1, 4), indicating that olfactory and/or visual inflorescence cues attract females of both mosquito species. To determine the contributing effect of tansy olfactory cues on mosquito attraction, we eliminated visual cues by occluding either intact inflorescences, or just their stems, with cheese cloth. Our findings that occluded intact inflorescences, but not just their stems, continued to attract both *A*. *aegypti* (z = 5.6, *P* < 0.0001) and *C*. *pipiens* (z = 10.9, *P* < 0.0001) (Fig. 1; Exps 2, 5) provide strong evidence that olfactory inflorescence cues suffice to attract females of both mosquito species. However, traps baited with a non-occluded intact inflorescence captured more female *A*. *aegypti* (z = 7.6, *P* = 0.014) and *C*. *pipiens* (z = 4.1, *P* < 0.0001) than traps fitted with an occluded intact inflorescence (Fig. 1; Exps 3, 6), revealing an additive effect between olfactory and visual inflorescence cues on mosquito attraction. We have obtained similar results with female *A*. *aegypti* and *C*. *pipiens* responding to non-occluded, or occluded, inflorescences of yarrow, *Achillea millefolium* (Supplementary Information: Fig. S1), suggesting that exploitation of di- or even multi-modal inflorescence cues by nectar-foraging mosquitoes may be a widespread phenomenon.

### Identification of Tansy Floral Odorants in Head Space Volatile (HSV) Extracts

**Figure 2:**
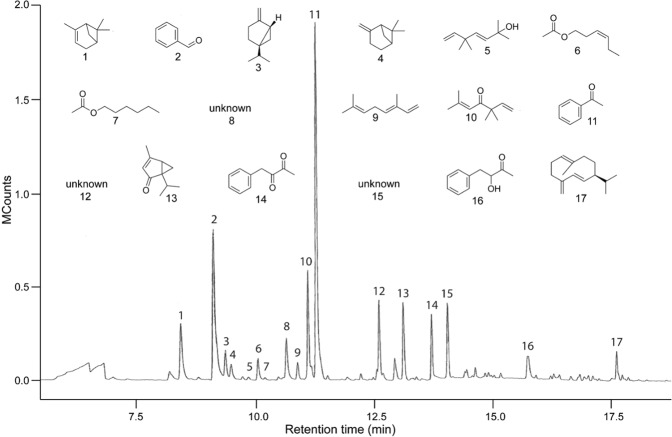
Headspace odorants of tansy inflorescences. 1 = (−)-α-pinene; 2 = benzaldehyde; 3 = (−)-sabinene; 4 = (−)-β-pinene; 5 = yomogi alcohol (2,5,5-trimethyl-3,6-heptadien-2-ol); 6 = (*Z*)-3-hexenyl acetate; 7 = hexyl acetate; 8 = unknown; 9 = (*E*)-β-ocimene (*trans*-3,7-dimethyl-1,3,6-octatriene); 10 = artemisia ketone (3,3,6-trimethyl-1,5-heptadien-4-one); 11 = acetophenone; 12 = unknown; 13 = umbellulone (4-methyl-1-(1-methylethyl)-bicyclo[4.1.0]hex-3-en-2-one); 14 = phenyl-2,3-butanedione; 15 = unknown; 16 = 3-hydroxy-4-phenyl-2-butanone; 17 = germacrene-D ((*E*,*E*)-1-methyl-5-methylene-8-(1-methylethyl)-1,6-cyclodecadiene).

To determine the olfactory cues that attract mosquitoes to tansy inflorescences, we captured floral head space volatiles (HSVs) and analyzed HSV extract by gas chromatography-mass spectrometry (GC-MS) (see Methods for detail). HSV extract contained 20 floral odorants (each > 1.25%) including acids, mono- and sesquiterpenes, ketones, alcohols and bifunctional compounds (Fig. 2). Drawing on these results, we prepared two types of synthetic blends for bioassays (Table 1; Supplementary Information): (a) a complete synthetic blend (CSB) containing all 20 odorants at 240 inflorescence-hour-equivalents (1 IHE = the amount of odorants released from one inflorescence during 1 h of odorant capture), a dose equivalent to the amount of inflorescence odorants emanating from one or two blooming tansy plants per hour for 24-h, and (b) a partial synthetic blend (PSB) of nine odorants comprising only those (butanoic acid, 2-methylpropionic acid, 2-methylbutanoic acid, 3-methylbutanoic acid, benzoic acid, hexanoic acid, (−)-α-pinene, benzaldehyde, acetophenone) also found in HSVs of human skin, breath, or skin microbiota (see Methods for detail).

**Table 1:** Headspace odorants and their absolute amounts present in 240 tansy inflorescence-hour-equivalents (1 IHE = the amount of odorants released from one inflorescence during 1 h of odorant capture) and tested in behavioral bioassays.

Compound	Amount (μg)	Human-shared?^1^	Supplier	Purity %
butanoic acid	2	Yes^12,13^	Sigma-Aldrich^b^	99
2-methylpropionic acid	1	Yes^12^	Sigma-Aldrich^b^	99
2-methylbutanoic acid	120	Yes^54^	Sigma-Aldrich^b^	98
3-methylbutanoic acid	240	Yes^12,13,54^	Sigma-Aldrich^b^	99
benzoic acid	2.5	Yes^53^	Sigma-Aldrich^b^	99.5
hexanoic acid	1	Yes^49,53,54^	Sigma-Aldrich^b^	99
(−)-α-pinene	13	Yes^55^	Sigma-Aldrich^b^	98
(−)-β-pinene	4	No	Sigma-Aldrich^b^	99
(−)-sabinene	7	No	Sigma-Aldrich^b^	75
(*E*/*Z*)-ocimene	5	No	Sigma-Aldrich^b^	>90
germacrene-D	7	No	Treatt^c,d^	40
benzaldehyde	43	Yes^53,55^	Sigma-Aldrich^b^	99
acetophenone	74	Yes^56^	Sigma-Aldrich^b^	99
artemisia ketone	23	No	Liberty Natural Products^d,e^	98
umbellulone	12	No	Sigma-Aldrich^b^	98
*(Z)*-3-hexenyl acetate	5	No	Sigma-Aldrich^b,f^	98
hexyl acetate	0.5	No	Sigma-Aldrich^b,f^	98
yomogi alcohol	0.5	No	Liberty Natural Products^d,e^	75
phenyl-2,3-butanedione	27	No	Gries-lab^g^	75
3-hydroxy-4-phenyl-2-butanone	8	No	Gries-lab^g^	25

Columns on the right indicate the commercial supplier and the purity of synthetic odorants. A 240-IHE synthetic blend dissolved in pentane/ether (1:1) was tested in bioassays.

^a^Numbers in parentheses correspond to literature references reporting these compounds in human headspace; ^b^Sigma-Aldrich (St. Louis, MO 63103, USA); ^c^Treatt Plc (Lakeland, FL 33805, USA); ^d^Liberty Natural Products (Portland, OR 97215, USA); ^e^see Supplementary Information for purification procedure; ^f^obtained by acetylation of corresponding alcohols; ^g^see Supplementary Information for synthetic procedures.

### Attractiveness of Tansy HSV Extract and Synthetic Floral Blends to Mosquitoes

**Figure 3:**
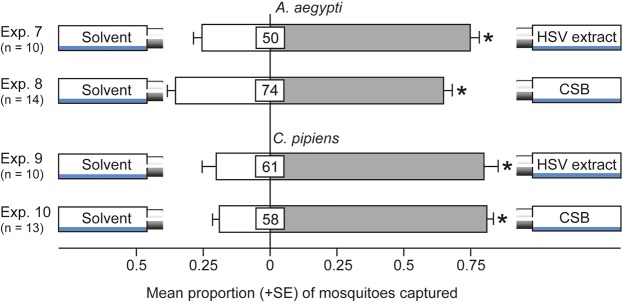
Effect of tansy headspace volatile extract and a synthetic blend of volatiles on mosquito attraction. Trap captures of 1- to 3-day-old female *A*. *aegypti* and 1- to 3-day-old female *C*. *pipiens* in response to tansy headspace volatile extract (HSV) and a complete synthetic blend (CSB) of these headspace volatiles (see Table 1). An asterisk indicates a significant preference (*P* < 0.0001) for the specific test stimulus (binary logistic regression analyses with logit link function); numbers within bars indicate the mean percentage of mosquitoes not captured.

**Figure 4:**
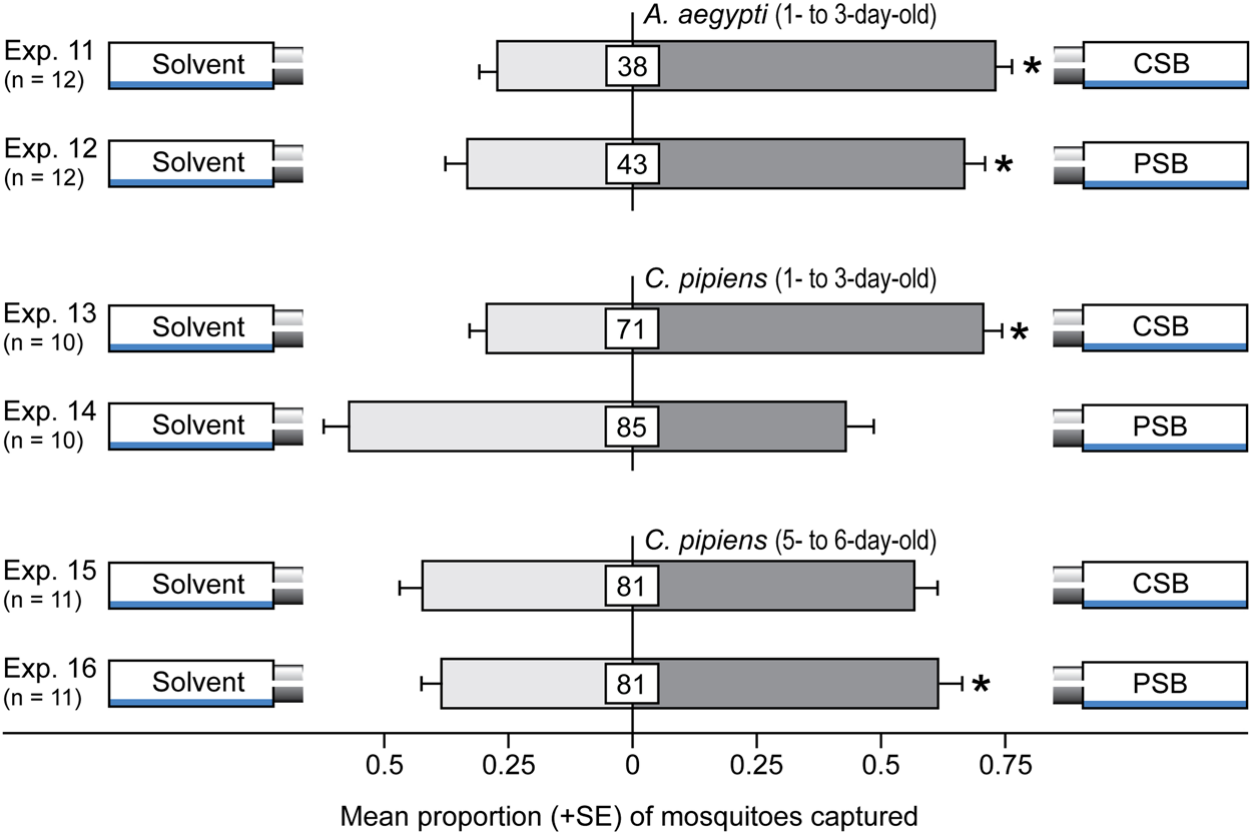
Effects of a complete synthetic blend of tansy headspace volatiles and a partial synthetic blend on mosquito attraction. Trap captures of 1- to 3-day-old female *A*. *aegypti* and 1- to 3-day-old or 4- to 6-day-old female *C*. *pipiens*. An asterisk indicates a significant preference (*P* < 0.05) for the specific test stimulus (binary logistic regression analyses with logit link function); numbers within bars indicate the mean percentage of mosquitoes not captured.

To determine whether the HSV extract was bioactive, we baited traps with aliquots of HSV extract and bioassayed them for attraction of mosquitoes. HSV-baited traps indeed captured more female *A*. *aegypti* (z = 7.4, *P* < 0.0001) and *C*. *pipiens* (z = 7.7, *P* < 0.0001) than corresponding control traps (Fig. 3; Exps 7, 9). Moreover, CSB-baited traps captured more female *A*. *aegypti* (z = 4.8, *P* < 0.0001) and *C*. *pipiens* (z = 9.9, *P* < 0.0001) than control traps (Fig. 3; Exps 8, 10), indicating that the CSB contained the critically important floral odorants that attracted mosquitoes to HSV extract or to the odor bouquet of intact inflorescences (Fig. 2, Table 1). To gauge the relative attractiveness of the CSB and the PSB, we tested them in sets of parallel experiments *versus* a solvent control. Taking into account that response preferences to the CSB (“floral nectar scent”) and the PSB (“vertebrate host scent”) may shift more strongly with aging female *C*. *pipiens* than with aging female *A*. *aegypti* that are aggressive daytime biters^39^, we tested groups of both young (1- to 3-day old) and old (4- to 5-day-old) female *C*. *pipiens*. As expected, CSB- and PSB-baited traps each captured more young female *A*. *aegypti* than control traps (z = 8.6, *P* < 0.0001; z = 5.5, *P* < 0.0001) (Fig. 4; Exps 11, 12). In contrast, only CSB-baited traps, but not PSB-baited traps, captured more young female *C*. *pipiens* than control traps (z = 4.7, *P* < 0.0001; z = −1.3, *P* = 0.2) (Fig. 4; Exps 13, 14). Conversely, PSB-baited traps, but not CSB-baited traps, captured more old female *C*. *pipiens* than control traps (z = 2.3, *P* = 0.02; z = 1.4, *P* = 0.17) (Fig. 4, Exps 15, 16). The combined data of experiments 13–16 reflect a resource preference shift from nectar to vertebrates by aging female *C*. *pipiens*. As predicted, PSB-baited traps failed to attract males of *A*. *aegypti* and *C*. *pipiens* that are not seeking vertebrate blood hosts (Supplementary Information: Fig. S2).

### Measurements of Tansy CO_2_ Emissions in the Field and Laboratory

**Figure 5:**
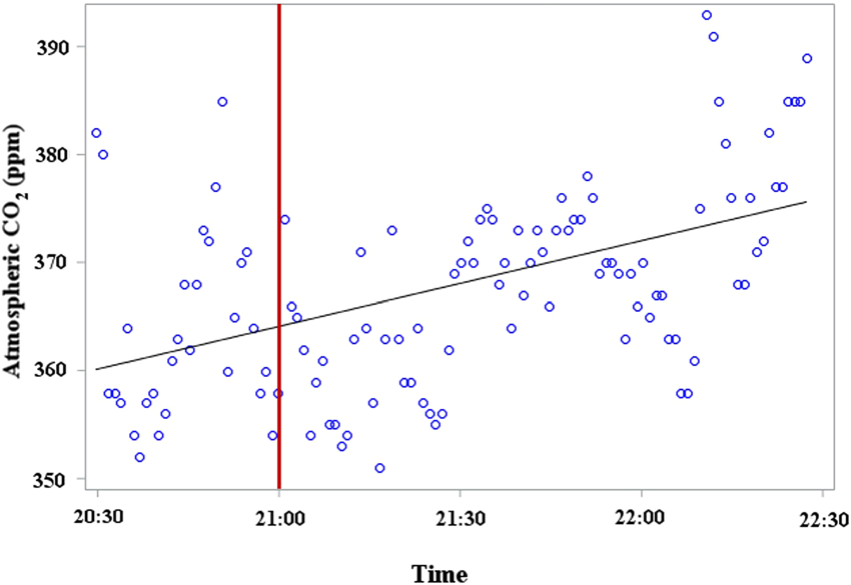
Changes in atmospheric CO_2_ concentration in a patch of tansies measured around dusk. The solid red line represents sunset, and the solid black line represents a linear fit (y = 360 + 8.24*hours, *P* = 0.0032, R^2^ = 0.6). Measured on 18 August 2015 in a patch of tansies, *Tanacetum vulgare*, in Burnaby, British Columbia.

To track changes in ambient CO_2_ around *in-situ* tansies, we placed an air quality monitor in a patch of tansies and took measurements from 20:30 to 22:30 h, with civil dusk occurring at circa 21:00 h. The ambient CO_2_ concentration in this patch significantly increased around civil dusk at a rate of 10.4 ppm hour^−1^ (Fig. 5). To measure CO_2_ emission directly from tansies, we field-collected a single inflorescence with 26 composite flowers during midday, inserted it into a water-filled vial, placed the vial in a 3.9-L Plexiglass chamber without natural light, inserted the monitor probe through a port in the chamber, and took CO_2_ measurements for four hours. During these measurements, the CO_2_ concentration increased at 1.19 ppm min^−1^ (Supplementary Information: Fig. S3), corresponding to 5 μL of CO_2_ min^−1^ emitted by the inflorescence.

### Effect of CO_2_ on Attraction of Mosquitoes

**Figure 6:**
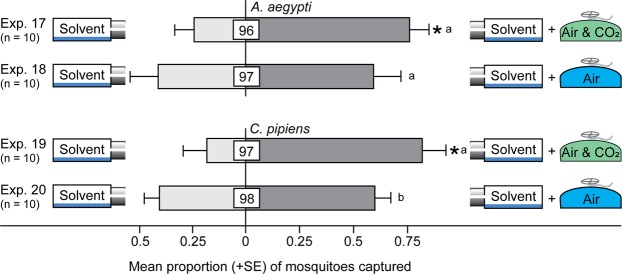
Effects of air or air containing CO_2_ on mosquito attraction. Trap captures of 1- to 3-day-old female *A*. *aegypti* and 1- to 3-day-old female *C*. *pipiens* in response to medical-grade air (air), or medical-grade air containing 1% CO_2_ (air & CO_2_). In each experiment, an asterisk indicates a significant preference (*P* < 0.05) for the specific test stimulus (binary logistic regression analyses with logit link function); different letters on paired bars in parallel experiments indicate a difference in the mean proportion of mosquitoes responding to respective stimuli (*P* < 0.05). Numbers within bars indicate the mean percentage of mosquitoes not captured (=non-responders); this percentage is relatively high here because experiments 17–20 were run for only 2 h, instead of 24 h (experiments 1–16).

To determine the effect of trace CO_2_ on attraction of mosquitoes, we ducted a flow of pure medical-grade air, or of medical-grade air enriched with 1% CO_2_, to the treatment traps in two parallel experiments. The flow of CO_2_-enriched air, but not of medical-grade air, afforded more trap captures of female *A*. *aegypti* (z = 2.3, *P* = 0.02; z = 0.7, *P* = 0.47) (Fig. 6; Exps 17, 18). The flow of CO_2_-enriched air, but not of medical-grade air, also afforded more trap captures of female *C*. *pipiens* (z = 2.11, *P* = 0.035; z = −0.5, *P* = 0.60) (Fig. 6; Exps 19, 20).

### Effect of Floral Odorants on Attraction of Mosquitoes to CO_2_

**Figure 7:**
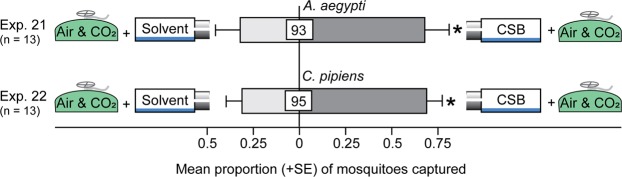
Additive effects of air containing CO_2_ and a complete synthetic blend of tansy headspace volatiles on mosquito attraction. Trap captures of 1- to 3-day-old female *A*. *aegypti* and 1- to 3-day-old female *C*. *pipiens* in response to medical-grade air containing 1% CO_2_ (air & CO_2_) and a complete synthetic blend (CSB) of tansy headspace volatiles (see Table 1). An asterisk indicates a significant preference (*P* < 0.05) for the specific test stimulus (binary logistic regression analyses with logit link function). Numbers within bars indicate the mean percentage of mosquitoes not captured (=non-responders); this percentage is relatively high because experiments 21–22 were run for only 2 h instead of 24 h (experiments 1–16).

To determine whether tansy floral odorants enhance the attractiveness of CO_2_ to mosquitoes, we fitted both the treatment and the control trap with a flow of CO_2_-enriched medical-grade air but baited only the treatment traps with the CSB. CO_2_-enriched medical-grade air in combination with the CSB afforded more trap captures of female *A*. *aegypti* (z = 2.4, *P* = 0.016) and *C*. *pipiens* (z = 2.1, *P* = 0.04) than CO_2_-enriched medical-grade air alone (Fig. 7; Exps 21, 22), indicating an interactive effect of CO_2_ and floral odorant cues on mosquito attraction.

## Discussion

Our data support the hypotheses that nectar-foraging females of *A*. *aegypti* and *C*. *pipiens*, analogous to host-foraging mosquito females, exploit a multimodal complex of CO_2_, semiochemical and visual floral cues, and that many key floral semiochemicals are shared with human hosts. To detect the (sometimes) subtle effects of the various floral cues, and to reveal interactions between them, it was imperative to run laboratory experiments where cues such as CO_2_ could be readily manipulated. Given that even intact inflorescences that represent all the cues of the entire inflorescence Gestalt attracted only 20–70% of the bioassayed mosquitoes (Fig. 1), less complex combinations of inflorescence cues – expectedly – afforded lower but still significant proportions of responding insects. Below, we elaborate on our findings and offer interpretations.

A 0.03-% rise in CO_2_ above ambient triggers host-seeking by mosquitoes^40^. Here we show that increasing CO_2_-levels approximating those around *in-situ* tansy inflorescences at dusk enhance attraction of mosquitoes to floral semiochemicals, thus demonstrating an interaction between bimodal inflorescence cues. Increased CO_2_ emissions from tansy inflorescences in our field patch at dusk (Fig. 5) may have been comparable to those from other nearby vegetation, and on their own may not have effectively guided nectar-foraging mosquitoes, but our laboratory experiments revealed that equivalent CO_2_ emissions enhance the attractiveness of inflorescence odorants to foraging mosquitoes (Fig. 7). Plant CO_2_ has previously been shown to affect insect-plant interactions^14^ but interactive effects were not investigated. For example, the haematophagous sand fly, *Phlebotomus papatasi*, locates sugar-rich plant tissue in response to differential CO_2_ emissions from various plant tissues, including those of the mosquito host plant *Ricinus communis*^21^. Similarly, CO_2_ respired by the bog orchid *Platanthera obtusata* is speculated to be a short-range foraging cue for its mosquito pollinators^4^. The tomato hornworm, *Manduca sexta*, exploits CO_2_ emissions from inflorescences of the sacred Datura, *Datura wrightii*, to locate its nectaries^14,20^. Moreover, larvae of the Western corn rootworm, *Diabrotica vergifera*, find corn roots based only on their CO_2_ emissions, and larvae of the cotton bollworm, *Helicoverpa armigera*, and the lesser cornstalk borer, *Elasmopalpus lignosellus*, orient towards CO_2_ sources, as do some tephritid fruit flies^14^.

Multimodal integration of CO_2_ and other sensory cues “drives” mosquito attraction to humans^10,11,16,41–45^ and may also underlie nectar-foraging by mosquitoes. CO_2_ on its own is attractive^46^, and as part of the human host cue complex is thought to (*i*) initiate mosquito take-off and flight^42–44^, (*ii*) enhance the attractiveness of host odorants at close range^10,11^, and (*iii*) to function as an activator that impels the mosquitoes’ responses to host semiochemical, visual, and thermal cues^15,16,41,45^. These concepts appear applicable to nectar-foraging by mosquitoes. As daylight fades, plants cease photosynthesis and become net producers of CO_2_. Concurrent release of CO_2_ from soil microorganisms, particularly in areas with plant roots^47^, contributes to a significant CO_2_ rise. Independent of photoperiod, some flowers even rhythmically produce elevated levels of CO_2_^48^. We posit that a CO_2_ rise from vegetated areas following dusk, or CO_2_ emitted from flowers, activates the mosquitoes’ responses to olfactory and possibly visual cues associated with nectar-producing inflorescences. If indeed a multimodal cue complex, rather than “just” a mono-modal cue, guides nectar-foraging mosquitoes to inflorescences, this would explain why in some reported studies floral extracts or synthetic floral odorants on their own were not effective in attracting mosquitoes.

The semiochemicals that tansy inflorescences share with human hosts suffice to attract female *A*. *aegypti* and aged (but not young) female *C*. *pipiens* (Fig. 4) that have apparently shifted from nectar- to host-foraging. Some of these shared semiochemicals [butanoic acid, 3-methylbutanoic acid, benzoic acid, hexanoic acid] that attract mosquitoes to human hosts and to tansy inflorescences are proven host-foraging cues for mosquitos^12,13,49–51^, whereas other compounds [e.g., acetophenone (but see^52^), 2-methylpropanoic acid] – while associated with humans^12,53–56^ – have yet to be rigorously tested in a host-seeking context. Interestingly, the human host odorants lactic acid and 1-octen-3-ol enhanced attraction of *Ae*. *aegypti* to fruit-based toxic sugar baits^57^ and admixture of human host odorants to plant-derived odorants increased attraction of some *Anopheles* spp^58^.

Our findings that the entire inflorescence Gestalt of olfactory and visual cues is more attractive to foraging mosquitoes than floral odorants alone (Fig. 1) indicate that visual displays contribute to the multimodal complex of inflorescence cues that attract mosquitoes to floral nectar. Even though human-visible floral colours may not affect mosquito foraging^2^, contrast within an inflorescence, or between an inflorescence and its surrounding^15^, may play a role. Moreover, ultraviolet floral reflections likely guide nectar-foraging mosquitoes, as shown in many other insect-pollinators^59^.

Haematophagy has arisen independently several times in the Insecta^60^, and phytophagy is one possible feeding habit from which haematophagy may have originated, at least for the Culicidae^61,62^. This previously postulated concept^60–62^ is supported by our findings that the same set of semiochemicals (PSB) attracts female mosquitoes to both tansy inflorescences and human hosts. An alternate explanation for shared cues between plants and vertebrates is that inflorescences “compete” with vertebrates for the attraction of mosquitoes, particularly >5-day-old, sugar-fed females that seem to prefer human-derived over nectar-derived odorants^63^.

We conclude that multimodal integration of CO_2_ and other sensory cues that drives mosquito attraction to humans appears to also drive mosquito attraction to inflorescences. Overlapping cues between plants and vertebrates support a previously postulated concept^60–62^ that haematophagy of some mosquito taxa may have arisen from phytophagy.

## Materials and Methods

### Rearing of Experimental Mosquitoes

For detailed information see Supplementary Information.

### Behavioural Bioassays

We ran all behavioural bioassays in mesh cages (77 × 78 × 104 cm) wrapped with black cloth except for the top to allow illumination from ambient fluorescent light. We kept cages at 23–26 °C, 40–60% RH, and a photoperiod of 14 L:10D. For each 24-h bioassay, we released 50 virgin, 1- to 3-day-old (unless otherwise stated), 24-h sugar-deprived females of *C*. *pipiens* or *A*. *aegypti* from a Solo cup (see Supplementary Information: Mosquito Rearing) into a cage. We randomly assigned the treatment and the control stimulus to adhesive-coated (The Tanglefoot Comp., Grand Rapids, MI 49504, USA), custom-made delta traps (9 cm × 15 cm) placed on each of two stands spaced 30 cm apart inside the cage. We wore latex gloves (Microflex Corporation, Reno, NV 89523, USA) during preparation of test stimuli.

### Effect of Olfactory and Visual Inflorescence Cues on Mosquito Attraction

We collected blooming inflorescences from *in-situ* tansies on the Burnaby campus of Simon Fraser University (SFU) and from potted, greenhouse-grown plants. The treatment stimulus consisted of one tansy inflorescence with 10–15 composite flowers cut from the plant bearing it. The control stimulus consisted of the stem of an inflorescence (with composite flowers excised and removed) cut from another plant. Because cut surfaces emanate “green leaf volatiles” and control plants had additional cuts due to the excision and removal of composite flowers, we inflicted cuts also on the stem of treatment plants. We covered all cut surfaces with petroleum jelly to minimize the release of green-leaf volatiles, inserted the treatment and the control plant into separate water-filled, parafilm-covered 4-mL vials, and placed each vial horizontally into a trap. We ran two experiments in parallel to rigorously study the effects of olfactory and visual inflorescence cues on mosquito attraction. To test the effect of olfactory cues, we occluded both the treatment and the control inflorescence by three layers of cheesecloth (VWR International, Radnor, PA 19087, USA) with a mesh size sufficiently wide to permit odorant dissemination. To test for an interactive effect between olfactory and visual cues, we occluded one inflorescence with three layers of cheesecloth and placed the other on top of the cheesecloth layers. To compare head-to-head the relative attractiveness of inflorescences presenting both visual and olfactory cues, or just olfactory cues, we occluded one of the two inflorescences with cheese cheesecloth.

### Capture and Attractiveness of Headspace Floral Odorants

We inserted 5–10 inflorescences into a 250-mL water-filled beaker which we then placed into a Pyrex® glass chamber (34 cm high × 12.5 cm wide). A mechanical pump drew charcoal-filtered air at a flow of 1 L min^−1^ for 24–72 h through the chamber and through a glass column (6 mm outer diameter × 150 mm) containing 200 mg of Porapak-Q™ adsorbent^64^. We desorbed floral odorants captured on Porapak-Q with 2 mL each of pentane and ether and bioassayed aliquots of Porapak-Q headspace volatile (HSV) extract for mosquito attraction. The treatment stimulus consisted of a 1-mL HSV extract aliquot [equivalent to the amount of odorants emanating from one or two blooming tansy plants per hour for 24 h, or approximately 240 inflorescence-hour-equivalents (IHE); 1 IHE = the amount of odorants released from one inflorescence during 1 h of odorant capture], emanating from a horizontally-placed 4-mL glass vial with a 2-mm hole in its lid. In the control stimulus, the HSV extract aliquot was replaced with the corresponding amount of pentane and ether (1:1 mix).

### Identification of Floral Odorants in HSV Extracts

After adding octyl acetate as an internal standard to HSV extract, we analyzed 2-µl aliquots by gas chromatography-mass spectrometry (GC-MS), operating a Saturn 2000 Ion Trap GC-MS fitted with a DB-5 GC-MS column (30 m × 0.25 mm i.d.; Agilent Technologies Inc., Santa Clara, CA 95051, USA) in full-scan electron impact mode. We used a flow of helium (35 cm s^−1^) as the carrier gas with the following temperature program: 50 °C (5 min), 10 °C min^−1^ to 280 °C (held for 10 min). The temperature of both the injector port and ion trap was 250 °C. To reveal the presence of low-molecular-weight carboxylic acids (which chromatograph poorly), we converted carboxylic acids to the corresponding silylated derivatives (which chromatograph well). To this end, we treated a 100-µL aliquot of HSV extract with BSTFA (10 µl; *N*,*O*-bis(trimethylsilyl)trifluoroacetamide) and TMCS (10%; trimethylchlorosilane; both Pierce Chemical Co., Rockford, IL 61101, USA) and after 5 min without any work-up analyzed 2-µl aliquots by GC-MS. We identified odorants in HSV extract by comparing their retention indices (RI; relative to *n-*alkane standards^65^) and their mass spectra with those reported in the literature^66^ and with those of authentic standards (Table 1).

### Preparation of a Synthetic Floral Odorant Blend

We prepared a synthetic blend of floral odorants (Table 1) including all those odorants present at >1.25% in floral HSV extract. The quantity and ratio of odorants in this synthetic blend matched those found in HSV extract. Moreover, we prepared a second synthetic blend (Table 1) consisting of only those floral odorants that are also found in headspace volatiles of human skin, breath, or skin microbiota.

### Attractiveness of Synthetic Floral Blends to Mosquitoes (1- to 3- or 5- to 6-day-old)

We tested the attractiveness of synthetic floral blends using the two-choice general bioassay design described above. In three sets of two parallel experiments, we tested a complete synthetic blend (CSB) of all floral odorants (Table 1) or a partial synthetic blend (PSB) comprising only those floral components also found in headspace volatiles of human skin, breath, or skin microbiota (Table 1) each *versus* a solvent control. We prepared the complete blend at approximately 240 IHEs dissolved in pentane/ether (1 mL; 1:1), and disseminated it from a horizontally-placed, 4-mL glass vial with a 2-mm hole in the lid. The control stimulus consisted of the equivalent solvent mixture (1 mL) disseminated from the same type of dispenser.

### Measurements of Tansy CO_2_ Emissions in the Field and Laboratory

We measured CO_2_ concentrations from a single cut tansy inflorescence weighing 3.6 g with a Q-Trak 7575-X air quality monitor (TSI Inc., Shoreview, MI 55126, USA) set to take readings every second and to average them in 1-min intervals. To track changes in ambient CO_2_ around *in-situ* tansies, we placed the monitor circa 5 cm above ground in a patch of tansies on the Burnaby campus of SFU, taking measurements from 20:30 to 22:30 h on 18 August 2015, with civil dusk occurring at circa 21:00 h.

### Effect of Trace CO_2_ on Mosquito Attraction

Using the two-choice general bioassay design described above, and running two experiments in parallel with both *C*. *pipiens* and *A*. *aegypti*, we tested the effect of CO_2_ on mosquito attraction. To provide a neutral stimulus, both traps in each experiment were fitted with a horizontally-placed, 4-mL glass vial containing pentane and ether (1 mL; 1:1) which were dispensed through a 2-mm hole in the lid. The test variable in one experiment consisted of a mixture of medical-grade air containing 1% CO_2_ (Praxair Inc., Mississauga, ON L5B 1M2, Canada) which amounts to a CO_2_ concentration about 10 × that near a single cut tansy inflorescence (see above), or comparable to that near a single intact tansy plant at the time when it is a net CO_2_ producer. To make sure that mosquitoes were not just responding to the flow of a gas mixture, the test variable in the parallel experiment consisted of medical grade air (Praxair Inc.). We delivered each test variable at the same flow rate [5000 μL min^−1^] through copper tubing (1.5 m × 2 mm i.d.) and aluminum tubing (0.5 m × 0.5 mm i.d.) to the respective delta trap and recorded the number of mosquitoes captured in each trap after 2 h.

### Effect of Tansy Floral Odorant Blend on Attraction of Mosquitoes to CO_2_

Using the two-choice general bioassay design described above, we tested whether floral odorants enhance attraction of *C*. *pipiens* and *A*. *aegypti* to CO_2._ In each experiment, we delivered a mixture of medical-grade air containing 1% CO_2_ to both the treatment and the control trap (as described above), baited the treatment trap with the complete blend of floral odorants (CSB; as described above), and fitted the control trap with a solvent control (as described above).

### Statistical Analyses of Data

We used SAS statistical software version 9.4 (SAS Institute Inc., Cary, NC 27513, USA) for data analyses, excluding from analyses experimental replicates with no mosquitoes responding. We used a binary logistic regression model with a logit link function and a Firth bias correction factor to compare mean proportions of responders between test stimuli, with overdispersion corrected for using the Williams method where appropriate (Exp. 9). We analyzed differences between experiments using non-adjusted least squares means. We worked with back-transformed data to obtain means and confidence intervals. We analyzed vegetative CO_2_ emission with autocorrelated linear regression to obtain concentration changes over time.

### Ethics approval and consent to participate

The research on plants performed in this study conforms with institutional, national, and international guidelines.

## Supplementary information

Supplementary Information

Supplementary Dataset 1

## Data Availability

Experimental data are presented in Supplementary Information: Experimental Data.
