# Author Correction: Multimodal floral cues guide mosquitoes to tansy inflorescences

**DOI:** 10.1038/s41598-019-44431-9

**Published:** 2019-05-24

**Authors:** Daniel A. H. Peach, Regine Gries, Huimin Zhai, Nathan Young, Gerhard Gries

**Affiliations:** 1 Department of Biological Sciences, Simon Fraser University, Burnaby, British Columbia V5A 1S6 Canada; 2 Present Address: Eurofins|Alphora Research Inc., Mississauga, Ontario L5K 1B3 Canada

Correction to: *Scientific Reports* 10.1038/s41598-019-39748-4, published online 07 March 2019

The original version of this Article contained a repeated typographical error in the Abstract and Introduction where,

“*Aedes aegpyti*”

now reads:

“*Aedes aegypti*”

This error has now been corrected in the PDF and HTML versions of the Article.

