# Supplementary material for: Multimodal floral cues guide mosquitoes to tansy inflorescences
Source: Sci Rep. 2019 Mar 7;9:3908. doi: 10.1038/s41598-019-39748-4 (PMC6405845; doi:10.1038/s41598-019-39748-4)

# Multimodal floral cues guide mosquitoes to tansy inflorescences

Daniel AH Peach[1,*], Regine Gries[1], Huimin Zhai[1,2], Nathan Young[1], Gerhard Gries[1]

(✉) Daniel Peach
dap3@sfu.ca

Gerhard Gries
gries@sfu.ca

[1]Department of Biological Sciences, Simon Fraser University, Burnaby, British Columbia V5A 1S6, Canada

[2]New address: Eurofins | Alphora Research Inc., Mississauga, Ontario, L5K 1B3, Canada

# Supplementary Information

**Mosquito Rearing**

**Preparative, Analytical and Synthetic Procedures**

**Supplementary Figure 1 -** Effect of olfactory and visual yarrow inflorescence cues on attraction female *A. aegypti* female *C. pipiens.*

**Supplementary Figure 2 -** Effect of the partial synthetic blend (PSB) on attraction of male *A. aegypti* and *C. pipiens*.

**Supplementary Figure 3 -** Quantification of $CO_2$ emission from a single tansy inflorescence.

**Supplementary Figure 4 -** Testing for side bias of the experimental design.

## Mosquito rearing

We reared *C. pipiens* and *A. aegypti* at 23-26 °C, 40-60% RH, and a photoperiod of 14L:10D. We maintained mixed groups of males and females in mesh cages (30 × 30 × 46 cm high) provisioned *ad libitum* with a 10-% sucrose solution. DP fed females once per week on his arm. For oviposition, gravid females were given access to water in a circular glass dish (10 cm diameter × 5 cm high) (*C. pipiens*) or in a 354-mL cup (Solo Cup Company, Lake Forest, IL 60045, USA) with paper towel lining (Kruger Inc., Montréal, QC H3S 1G5, Canada) (*Ae. aegpyti*). We transferred egg rafts of *C. pipiens* to water-filled trays (45 × 25 × 7 cm high), and paper towel strips with *A. aegypti* eggs to circular glass dishes (10 cm diameter × 5 cm high) containing water and brewers yeast (U.S. Biological Life Sciences, Salem, MA 01970, USA). Two to four days later, we transferred the dish contents to water-filled trays (45 × 25 × 7 cm high). We provisioned larvae with NutriFin Basix tropical fish food (Rolf C. Hagen Inc., Baie-D'Urfe, QC H9X 0A2, Canada), and transferred pupae via a 7-mL plastic pipette (VWR International, Radnor, PA 19087, USA) to water-containing 354-mL Solo cups covered with a mesh lid. We collected eclosed adults via aspirator and placed them in similar cups, along with a cotton ball soaked in a 10-% sucrose solution.

## Preparative, Analytical and Synthetic Procedures

### Purification of Germacrene-D

Germacrene-D was purified to 93% from Treatt Plc (Lakeland, FL 33805, USA) (40% technical grade) by high-performance liquid chromatography (HPLC) [Waters HPLC system (600 Controller, 600 Delta Pump, 2487 Dual Lamda Absorbance Detector [Waters Corp. Milford, MA  01757 USA], using a $C_{18}$ reversed phase column (Synergi-Hydro, 250 × 60 mm, 4 µ) eluted with acetonitrile (1 ml/min).

### Purification of Artemisia ketone

Artemisia ketone (3,3,6-trimethyl-1,5-heptadien-4-one) is present (34%) in Wormwood (*Artemisia annua*) essential oil (Liberty Natural Products, Portland, OR 97215, USA) and was isolated (190 g, 98% pure) by repetitive silica gel column chromatography using hexane and ethyl acetate (90:10) as eluents.

### Purification of Yomogi alcohol

Yomogi alcohol (2,5,5-trimethyl-3,6-heptadien-2-ol) is present (15%) in Chamomile Morocco (*Ormensis multicaulis*) essential oil (Liberty Natural Products) which also contains Santolina alcohol (62%) as well as cineole and monoterpenes. Yomogi alcohol was isolated from 3 g of the essential oil by flushing analyte twice through a silica gel column using hexane and ethyl acetate as eluents (first flash: 80:20; second flash: 85:15). This procedure yielded 180 mg of Yomogi alcohol (50% pure) which was then further purified by HPLC (see above) using acetonitrile and water (60:40; 1 ml/min) as eluents. This purification procedure resulted in a mixture of Yomogi alcohol (75%) and Santolina alcohol (25%) as by-product.

### Preparation of 1-phenylbutane-2,3-dione and 1-phenyl-3-hydroxy-2-butanone

A solution of DL-3-phenyllactic acid (166 mg, 1.0 mmol, 1.0 eq.) in $CH_2Cl_2$ (15 ml) was sequentially added to *N*,*O*-dimethylhydroxylamine hydrochloride (195 mg, 2.0 mml, 2.0 eq.) and 1,1'-carbonyldiimidazole (324 mg, 2.0 mml, 2.0 eq.) at 0 °C. After 30 min, the reaction was stirred at ambient temperature overnight before quenching with water. The aqueous layer was separated and extracted with $CH_2Cl_2$ (15 mL). The combined organic layer was washed sequentially with 10 % HCl aqueous solution, 5% $NaHCO_3$ aqueous solution and brine, then dried over $MgSO_4$ and concentrated. The residue was used for the next step without further purification. The prepared above Weinreb amide was dissolved in anhydrous THF (8 ml) and cooled to -78 °C. MeMgBr (3.0 M in $Et_2O$, 0.6 ml, 1.8 mmol, 1.8 eq.) was added, and the mixture was stirred at 0 °C for 5 h before quenching with saturated aqueous $NH_4Cl$ (5 mL). The aqueous layer was separated and extracted with EtOAc (10 mL). The combined organic layer was washed sequentially with water and brine, then dried over $Na_2SO_4$ and concentrated. The residue was purified by flash chromatography (hexane/EtOAc, 6/1) yielding 87 mg (53% over 2 steps) of 1-phenyl-3-hydroxy-2-butanone as colourless oil. A sample of this ketone-alcohol (49 mg, 0.3 mmol, 1.0 eq.) was dissolved in dry $CH_2Cl_2$ (10 ml), and $NaHCO_3$ (38 mg, 0.45 mmol, 1.5 eq.) was added followed by Dess-Martine periodinane (190 mg, 0.45 mmol, 1.5 eq.). After stirring the reaction mixture at ambient temperature for 1 h, the mixture was treated with a 10% aqueous solution of $Na_2S_2O_3$ (5 ml) and a saturated aqueous solution of $NaHCO_3$ (5 mL) and then stirred for an additional 20 min. The aqueous layer was separated and extracted with EtOAc (3× 10 ml). The combined organic layers were washed with brine (10 ml), dried over magnesium sulfate and concentrated. Purification by flash chromatography (hexane/ethyl acetate, 10/1) provided 65 mg (75 % pure) of the diketone as a yellow oil.

**Figure S1. Effect of olfactory and visual yarrow inflorescence cues on attraction female *A. aegypti* female *C. pipiens*.** Two-choice laboratory experiments with a paired-trap design demonstrated that traps baited with a non-occluded yarrow inflorescence captured more female *A. aegypti* ($z = 4.3$, $P < 0.0001$) and *C. pipiens* ($z = 10.6$, $P < 0.0001$) than traps fitted with a non-occluded stem of an inflorescence (Exps. S1, S4), indicating that olfactory and/or visual inflorescence cues attracted females of both mosquito species to yarrow. We determined the contributing effect of yarrow olfactory cues on mosquito attraction via eliminating visual cues by occluding either intact inflorescences, or just stems, with cheese cloth. Our findings that occluded inflorescences, but not just their stems, continued to attract both *A. aegypti* ($z = 4.8$, $P < 0.0001$) and *C. pipiens* ($z = 7.8$, $P < 0.0001$) (Exps. S2, S5) provided further evidence that olfactory cues of yarrow inflorescence attract females of both mosquito species. However, traps baited with a non-occluded inflorescence captured more female *A. aegypti* ($z = 3.2$, $P < 0.001$) and *C. pipiens* ($z = 3.4$, $P < 0.01$) than traps with an occluded inflorescence (Exps. S3, S6), revealing additive attraction of mosquitoes to yarrow olfactory and visual inflorescence cues. These findings confirm our results with tansies that nectar-foraging mosquitoes exploit multimodal inflorescence cues. A rectangular box with hatched lines indicates that the occluded inflorescence offered no visual cues. An asterisk indicates a significant preference ($P < 0.001$) for the test stimulus (binary logistic regression analyses with logit link function); different letters on paired bars in parallel experiments indicate a difference in the mean proportion of mosquitoes responding to respective stimuli ($P < 0.05$); numbers within bars indicate the mean percentage of mosquitoes not captured.

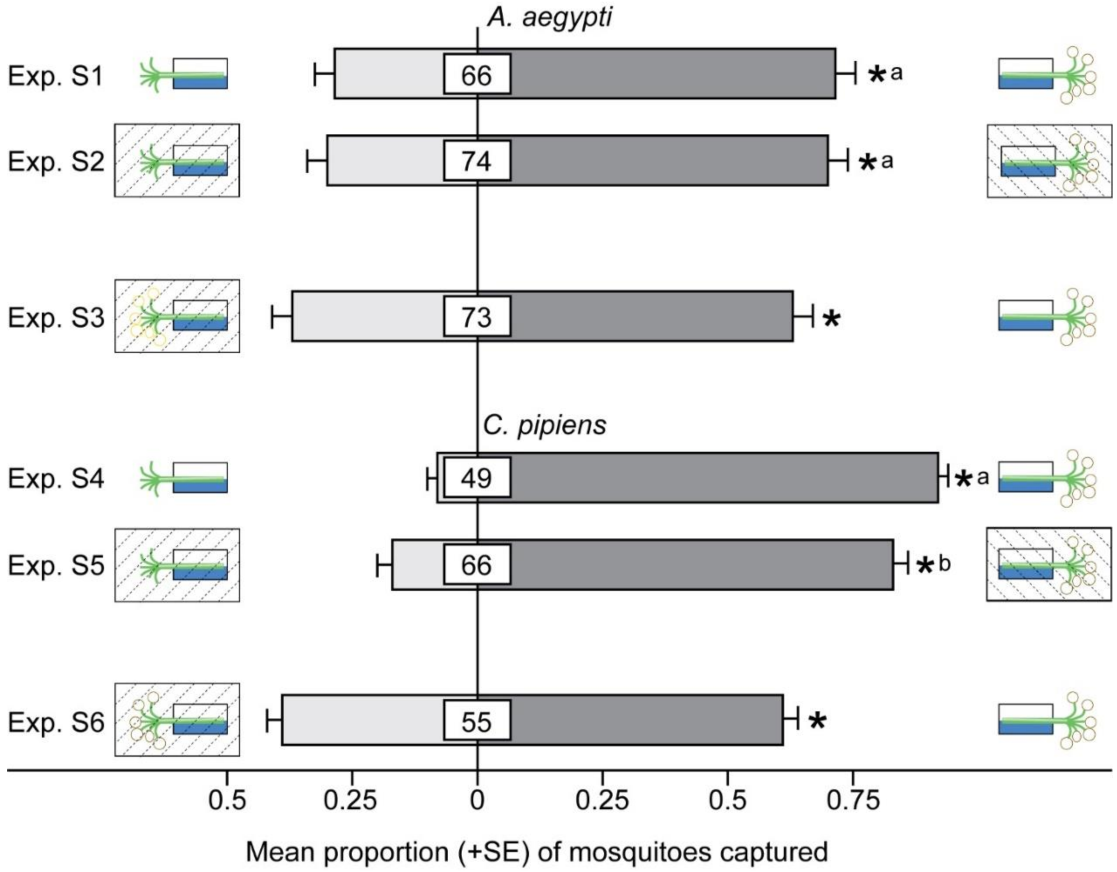

**Figure S2. Effect of the partial synthetic blend (PSB) on attraction of male *A. aegypti* and *C. pipiens*.** Two-choice experiments revealed that the PSB was not attractive to male *A. aegypti* (z = 0, *P* = 1) or to male *C. pipiens* (z = 0.62, *P* = 0.54) when compared to blank solvent (Exps S7, S8). These results suggest that the PSB was not a floral attractant. Numbers within bars indicate the mean percentage of mosquitoes not captured.

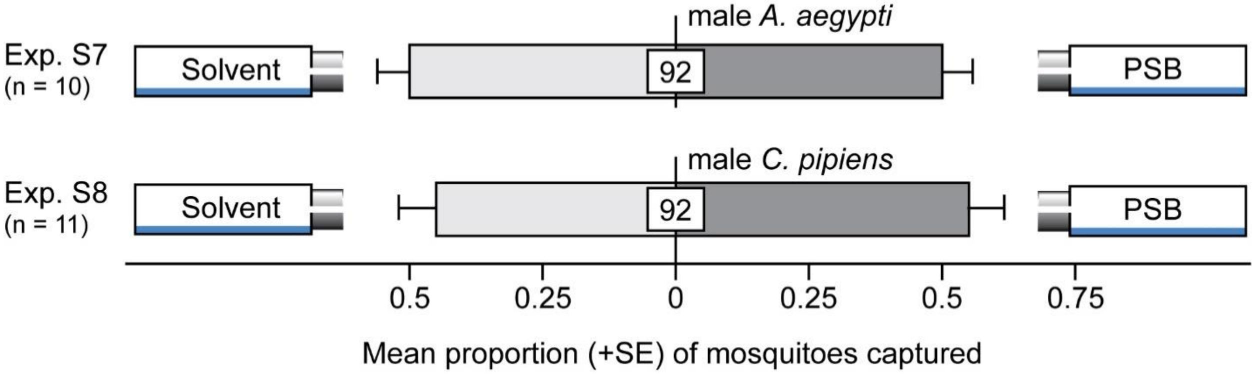

**Figure S3. Quantification of $CO_2$ emission from a single tansy inflorescence.** Increase in atmospheric $CO_2$ measured every minute from a single tansy inflorescence (3.63 g) enclosed in a 3.9-L container. The solid black line represents an autocorrelated linear fit (y = 303 + 74.88*hours, $P < 0.0001$, $R^2 = 0.999$), corresponding to $CO_2$ increase of approximately 5 µL $min^{-1}$. We measured $CO_2$ concentrations with a Q-Trak 7575-X air quality monitor (TSI Inc., Shoreview, MI 55126, USA) set to take readings every second and to average them in 1-min intervals.

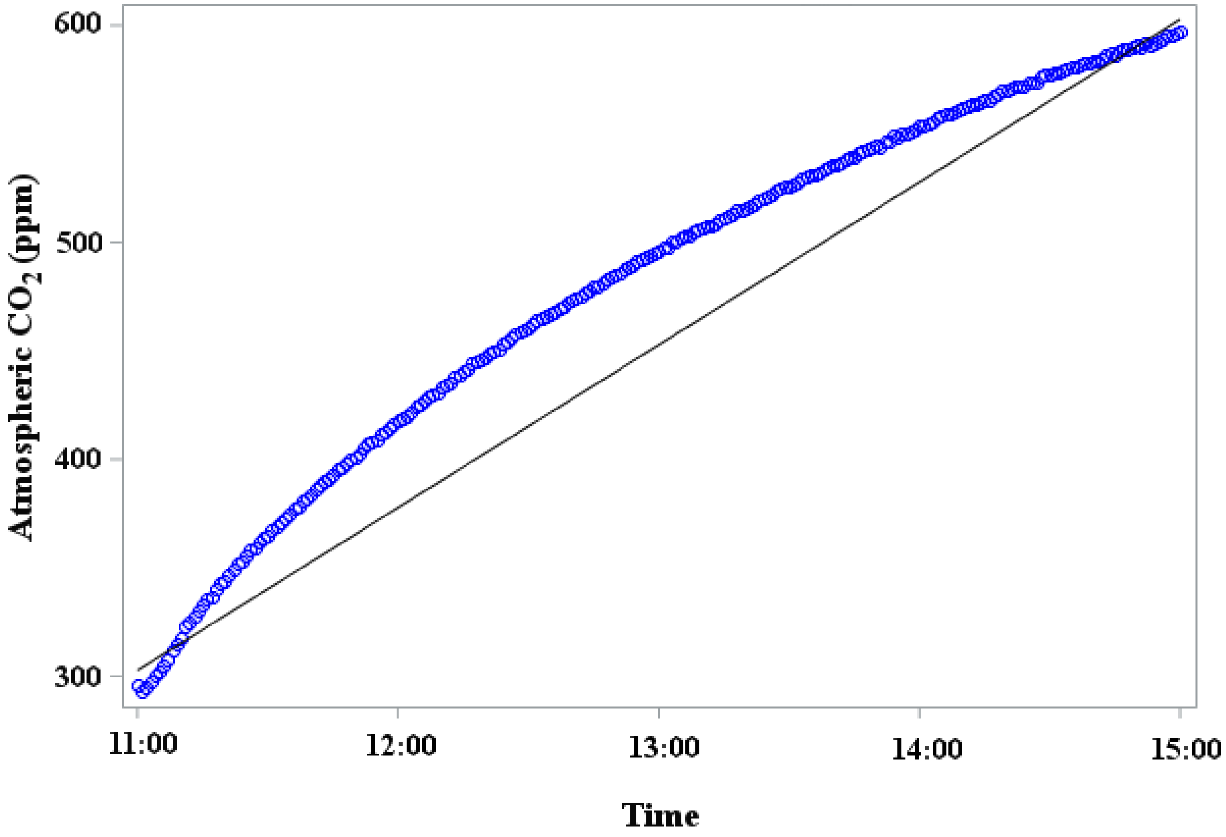

**Figure S4. Testing for side bias of the experimental design.** Paired tests run in parallel with blank traps for 24 h with female *C. pipiens* revealed no effect of side-bias in test cage A (z= -0.35, p = 0.72) or in test cage B (z = -0.21, *P* = 0.83), and no significant difference between the two cages (z= -0.07, p= 0.94) (Exps S9, S10).

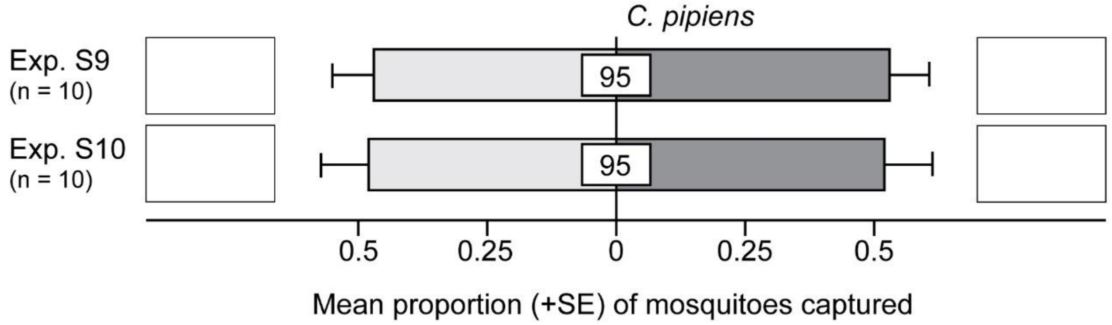

Supplement: Supplementary file 1 — Supplementary Information [file 41598_2019_39748_MOESM1_ESM.pdf]
